# An in-depth assessment of India’s Mother and Child Tracking System (MCTS) in Rajasthan and Uttar Pradesh

**DOI:** 10.1186/s12913-015-0920-2

**Published:** 2015-08-11

**Authors:** Rajeev Gera, Nithiyananthan Muthusamy, Amruta Bahulekar, Amit Sharma, Prem Singh, Amrita Sekhar, Vivek Singh

**Affiliations:** Public Health Foundation of India, 14, Community Centre, Panchsheel Park, New Delhi, 110017 India

## Abstract

**Background:**

India’s Mother and Child Tracking System (MCTS)^1^ is an information system for tracking maternal and child health beneficiaries in India’s public health system, and improving service delivery planning and outcomes. This ambitious project was launched in 2009 and currently covers all states in India, but no in-depth assessment of the system has been conducted. This study by the Public Health Foundation of India (PHFI) evaluated the performance of MCTS and identified implementation challenges in areas in Rajasthan and Uttar Pradesh (UP) in December 2012.

**Methods:**

Two assessment methods were employed: a Data Quality Assessment (DQA) to evaluate data quality and an assessment survey to identify implementation challenges. The survey comprised semi-structured questionnaires for health staff in the sampled districts, observation checklists and survey investigator notes. Purposive sampling was used for selecting two districts in each state and two blocks in each district. For the DQA, 45 mothers who became pregnant and 84 children born within the stipulated timeframes were randomly sampled.

**Results:**

DQA overall performance numbers were 34 % for pregnant women and 33 % for children in the Rajasthan study areas, while UP’s performance numbers were 18 % for pregnant women and 25 % for children. Weaknesses in the MCTS' data completeness accounted for much of this performance shortfall. The beneficiary profiles for Rajasthan were largely incomplete, and the MCTS in UP struggled to register beneficiaries. Shared challenges in both states were the absence of clear processes and guidelines governing data processes, and the lack of systematic monitoring and supervision frameworks for MCTS implementation. As a result, Front Line Health Workers (FHWs) were overburdened with data documentation work, and there were long delays in data capturing. FHWs and block level health officials were not adequately trained in using the MCTS. UP staff reported unreliable internet and electricity availability, lack of dedicated data entry personnel, and a shortage of consumables such as MCTS registers.

**Conclusions:**

There is an urgent need to create data processes and supervision guidelines that complement existing workflows and service delivery priorities. Health staff should be trained to implement these guidelines. MCTS outputs, such as service delivery planning tools, should replace existing tools once data quality improves.

## Background

Among the most vulnerable populations, pregnant women and children in resource-poor settings need health systems that are capable of delivering timely and quality care. At the forefront of service delivery in many Low and Middle Income Countries (LMICs) are Frontline Health Workers (FHWs), who are responsible for identifying all eligible beneficiaries within their catchment areas and ensuring that each beneficiary receives the full schedule of services.

Robust Health Information Systems (HISs), powered by complete and accurate data, can play a powerful role in facilitating these routine service delivery activities by FHWs [1], while also improving decision making by supervisory and managerial health officials [2]. Recognizing this, the World Health Assembly in 2005 endorsed e-health, of which HISs are a core component, as a means of strengthening health systems [3].

Since then, many LMICs have institutionalised HISs to improve and inform the implementation of public health programs [4], with some of them opting to include Information Communication Technologies (ICTs) into their health systems for data management [5]. There has been some preliminary evidence from Brazil that their HIS has improved the efficiency of outpatient services and increased patient inflow [4]. A similar intervention for mothers and children in Sau Paulo reports impressive improvements in the coverage of Maternal and Child Health (MCH) services [6].

However, data quality challenges in LMICs may hamper the usefulness of such systems in achieving better health outcomes [4]. There has been a dearth of systematic evaluations to gauge the data quality and implementation effectiveness of HISs [7, 8]. The few HIS assessments that have been carried out in the developing world have demonstrated usefulness in not only highlighting HIS implementation challenges, but also in pointing the way towards implementation improvement measures [9–12].

The Mother and Child Tracking System is a beneficiary-specific database for MCH services delivered through the Indian public health system. It was launched in 2009 as part of a global trend towards harnessing e-health innovations in improving service delivery, and also because India’s existing Health Management Information System (HMIS) was not meeting the service delivery needs of FHWs [1]. As a Mission Mode Project (MMP) under the Government of India’s National e-Governance Plan (NeGP), MCTS has clearly defined “objectives, scopes, and implementation timelines and milestones, as well as measurable outcomes and service levels” [13]. The MCTS is designed to capture and track all pregnant women (from conception up to 42 days post-partum) and all new-born children (up to 5 years of age). Its objectives are to ensure that [14]:

- all pregnant women receive their full Antenatal Care (ANC) and Postnatal Care (PNC) services at the due times;
- institutional deliveries for pregnant women, particularly for high risk mothers, are encouraged; and
- all children receive the full immunisation schedule at the due times.

**Fig. 1:**
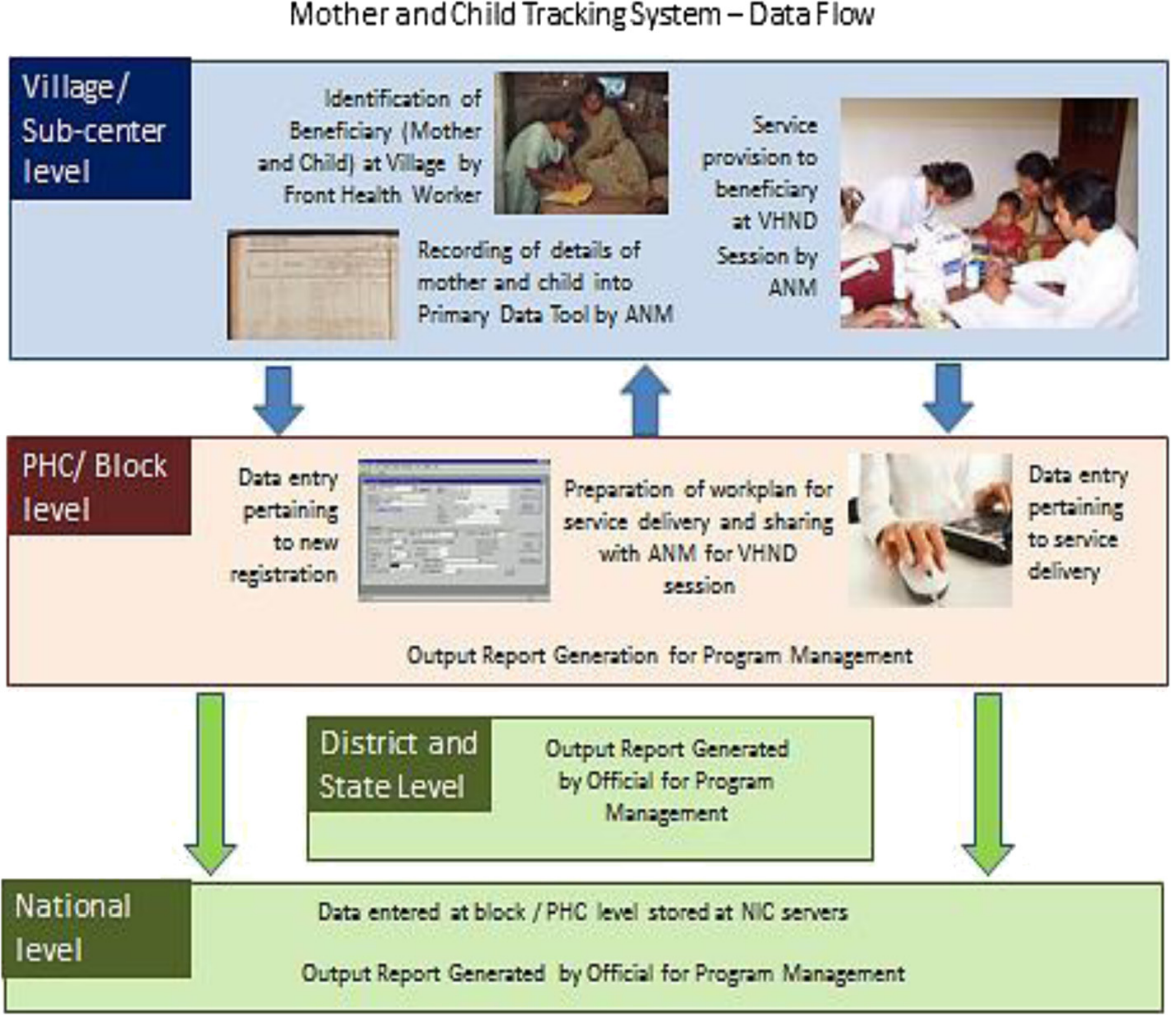
Mother and child tracking system: data flow

Beneficiary and service delivery data are written by FHWs on registers and formats and then transferred to the nearest primary health centre (PHC) for entry into the MCTS portal by data entry operators (DEOs). All health facilities, from the state to the most peripheral health sub-centres (HSCs), are mapped in the portal, which also maps FHWs to specific HSCs. The data enables the MCTS to generate workplans for FHWs, detailing forthcoming service delivery needs, such as antenatal check-ups or immunization sessions, on a per-beneficiary basis. Supervisory officials can also generate reports from the MCTS web portal that indicate MCTS performance (beneficiary registration rates) or service delivery performance (e.g. percentage of children fully immunized). Figure 1 presents the flow of data into the MCTS from the field level and the MCTS output reports generated for service delivery, management and supervisory officials.

As can be noticed from Fig. 1, the success of the MCTS as a data system relies heavily on processes and practices at the village/ HSC level. The field-level data collection, consolidation and transfer activities ultimately determine MCTS data quality. Preliminary findings from rapid desk reviews of MCTS national aggregate data have indicated low performance levels for this ambitious, but much needed project. Data acquired from the MCTS cell in July 2012 indicated that relative to the estimated beneficiary population numbers, registration rates stood at 63 % for pregnant mothers, and 59 % for children. Wide discrepancies were noticed between service delivery rates in the HMIS and MCTS portals.

These indications of poor MCTS performance, coupled with the potential of the MCTS to ensure timely MCH service delivery, underscored the need for the first ever in-depth assessment of the MCTS. In December 2012, PHFI carried out the assessment in four districts spread over two large states, Rajasthan and Uttar Pradesh, to identify the root causes of the low performance levels.

## Methods

### Goals, objectives and methods

The goals of the assessment were to assess the implementation challenges of the MCTS as an information system in the study areas and to propose an approach for strengthening the system. The objectives were as follows:

- Carry out a data quality assessment (DQA) on the data found in the MCTS portal using registers and formats used by FHWs as the primary data source for data quality evaluation.
- Use an assessment survey with key government officials to identify processes, practices, infrastructural set-up, budgeting conditions and human resource situations related to MCTS implementation.
- Utilize results from the DQA and assessment survey to identify implementation challenges and propose an approach for strengthening the system

The methodology for the MCTS assessment was divided into two key areas, namely a DQA, and an assessment survey. DQAs have been internationally recognized and are conducted globally to evaluate the data integrity of information systems. The World Health Organisation (WHO), Gavi (a private-public Global Vaccine Alliance) and the Global Fund have recommended detailed DQA approaches [15–17]. Our DQA was meant to provide completeness, accuracy and overall performance indicators for the MCTS data of sampled beneficiaries. While the DQA findings provided quantitative indicators on the data integrity of the sampled beneficiaries’ MCTS data, they did not unpack the implementation dynamics and root causes behind those numbers. Therefore, the DQA was supplemented by an assessment survey in which all staff members involved in MCTS implementation were interviewed to understand implementation challenges and bottlenecks. In other words, the assessment survey contextualized the DQA findings by uncovering field implementation dynamics which indicate the root causes of data quality weaknesses or strengths. In combination, these two methods allowed for a comprehensive analysis of field implementation realities.

### Sampling

This study was conducted in partnership with program managers in the Ministry of Health and Family Welfare (MoHFW), Government of India (GoI), who requested input into the sampling design. At their suggestion, the geographical scope of the assessment was two districts each in Rajasthan and UP, two blocks (Primary Health Care Centres at the block level) in each district, with two HSCs in each block. Rajasthan and UP were selected as Rajasthan implemented the MCTS before it was launched across the country, and UP has the largest population in India. In the selection of districts within each study state, indicators that represent both MCTS and health system performance were used to stratify districts into ‘good’ and ‘poor’ performing categories. The indicators used for the stratification of districts were:

- percentage of health facilities not reporting mothers’ information in the MCTS web portal in July 2012;
- percentage of pregnant women who had received their second ANC service in April 2012; and
- percentage of children born in April 2012 who had received BCG vaccination by July 2012

Six districts in Rajasthan (three performing well, three performing poorly), and five districts in Uttar Pradesh (two performing well, three performing poorly) were shortlisted for selection in collaboration with the MoHFW. With active inputs from the Ministry, one district from each category was selected for each state.

The indicators for the shortlisted districts are presented in Table 1, and the selected districts are bolded. The names of the districts have been hidden to protect study participant identities. Two blocks in each district and two HSCs in each block were selected in consultation with district- and block-level officials.

**Table 1:** Shortlisted districts for the MCTS assessment

State	District	% of health facility not reporting mother’s information	% of pregnant women who received second ANC - April 2012	% of children born in April 12 who received BCG vaccine	Criteria match
Rajasthan	A	4.44	45	43	Good performing
	B	9.68	49	50	Good performing
	C	20.97	4	37	Poor performing
	D	25.45	12	26	Poor performing
	E	8.65	7	30	Poor performing
	F	5.71	22	73	Good performing
Uttar Pradesh	G	0.00	10.2	43.4	Good performing
	H	44.44	8.8	65.1	Poor performing
	I	57.89	32.2	48.8	Poor performing
	J	68.25	17.4	37.7	Poor performing
	K	30.77	27.3	54.6	Good performing

As one of the parameters for this study was data quality, a DQA was carried out in each of the districts for a random sample of pregnant women and children in December 2012. The target sample size was six pregnant women who were eligible for MCTS registration from July 2011 to December 2011, and 12 children who were eligible for MCTS registration from July 2011 to March 2012, per block. This amounts to 12 pregnant women and 24 children targeted from each of the study districts, and 24 pregnant women and 48 children targeted from each state. The rationale for selecting pregnant women and children in the stipulated time period was to ensure that all sampled beneficiaries were eligible to have received all scheduled vaccines under India’s Universal Immunization Program (UIP). The target sample size was determined by feasibility considerations: the team had to manage data collection, entry, cleaning and report generation in a time-bound manner, and the workload from this sample size was manageable. It was also decided that children’s data should be prioritized, leading the study team to sample more children.

A total of 21 women and 40 children were sampled in Rajasthan, while 24 women and 44 children were sampled in UP. It is important to note that this sampling was not intended to produce representative nationwide or statewide MCTS data quality results, but to generate data quality indicators to complement the assessment survey.

### DQA indicators and data collection

The DQA assessed MCTS data in three areas–completeness, recording accuracy and overall performance–using six indicators. The DQA exercise was guided by the Global Fund’s Data Quality Audit Tool [16] and the Immunization Data Quality Assessment (IDQA) tool from WHO [15]. However, the operational definitions for the selected indicators were modified and contextualised according to the requirements of this assessment. Some indicators were newly devised. Table 2 lists the indicators and the mathematical equations used to calculate them.

**Table 2:** Operational definitions for the DQA indicators

Sr. No.	Description of the indicator	Equations
Completeness		
1	Percentage of beneficiary profiles found missing in MCTS portal	$\frac{\mathrm{No}.\ \mathrm{of}\ \mathrm{beneficiary}\ \mathrm{profiles}\ \mathrm{missin}{\mathrm{g}}_{\mathrm{j}}}{\mathrm{Total}\ \mathrm{sampled}\ \mathrm{beneficiarie}{\mathrm{s}}_{\mathrm{j}}}\times 100$
2	Percentage of total data fields found with entries, in the primary data source and the MCTS portal respectively	$\frac{\mathrm{Total}\ \mathrm{no}.\ \mathrm{of}\ \mathrm{data}\ \mathrm{field}\mathrm{s}\ \mathrm{with}\ \mathrm{entrie}{\mathrm{s}}_{\mathrm{ij}}}{\mathrm{Total}\ \mathrm{no}.\ \mathrm{of}\ \mathrm{data}\ \mathrm{field}{\mathrm{s}}_{\mathrm{ij}}}\times 100$
3	Percentage of common data fields found empty in both the primary data source and MCTS portal	$\frac{\mathrm{Total}\ \mathrm{no}.\ \mathrm{of}\ \mathrm{common}\ \mathrm{data}\ \mathrm{fields}\ \mathrm{found}\ \mathrm{empty}\ \mathrm{In}\ \mathrm{both}\ \mathrm{primary}\ \mathrm{data}\ \mathrm{source}\ \mathrm{and}\ \mathrm{MCTS}\ \mathrm{porta}{\mathrm{l}}_{\mathrm{j}}}{\mathrm{Total}\ \mathrm{no}.\ \mathrm{of}\ \mathrm{common}\ \mathrm{data}\ \mathrm{fields}\ \mathrm{in}\ \mathrm{both}\ \mathrm{primary}\ \mathrm{data}\ \mathrm{source}\ \mathrm{and}\ \mathrm{MCTS}\ \mathrm{porta}{\mathrm{l}}_{\mathrm{j}}} \times 100$
4a	Percentage of common data fields with entry in MCTS portal, without entry in primary data source	$\frac{\mathrm{Total}\ \mathrm{no}.\ \mathrm{of}\ \mathrm{common}\ \mathrm{data}\ \mathrm{fields}\ \mathrm{with}\ \mathrm{e}\mathrm{ntry}\ \mathrm{in}\ \mathrm{MCTS}\ \mathrm{porta}\mathrm{l},\ \mathrm{with}\mathrm{out}\ \mathrm{e}\mathrm{ntry}\ \mathrm{in}\ \mathrm{primary}\ \mathrm{data}\ \mathrm{sourc}{\mathrm{e}}_{\mathrm{j}}}{\mathrm{Total}\ \mathrm{no}.\ \mathrm{of}\ \mathrm{common}\ \mathrm{data}\ \mathrm{fields}\ \mathrm{in}\ \mathrm{both}\ \mathrm{primary}\ \mathrm{data}\ \mathrm{sourc}\mathrm{e}\ \mathrm{and}\ \mathrm{MCTS}\ \mathrm{porta}{\mathrm{l}}_{\mathrm{j}}}\times 100$
4b	Percentage of common data fields with entry in primary data source, without entry in MCTS portal	$\frac{\mathrm{Total}\ \mathrm{no}.\ \mathrm{of}\ \mathrm{common}\ \mathrm{data}\ \mathrm{fields}\ \mathrm{with}\ \mathrm{entry}\ \mathrm{in}\ \mathrm{primary}\ \mathrm{data}\ \mathrm{source},\ \mathrm{with}\mathrm{out}\ \mathrm{entry}\ \mathrm{in}\ \mathrm{MCTS}\ \mathrm{porta}{\mathrm{l}}_{\mathrm{j}}}{\mathrm{Total}\ \mathrm{no}.\ \mathrm{of}\ \mathrm{common}\ \mathrm{data}\ \mathrm{fields}\ \mathrm{in}\ \mathrm{both}\ \mathrm{primary}\ \mathrm{data}\ \mathrm{source}\ \mathrm{and}\ \mathrm{MCTS}\ \mathrm{porta}{\mathrm{l}}_{\mathrm{J}}} \times 100$
Accuracy		
5	Percentage of common data fields with matching entries in both primary data tool and MCTS portal	$\frac{\mathrm{Total}\ \mathrm{no}.\ \mathrm{of}\ \mathrm{common}\ \mathrm{data}\ \mathrm{fields}\ \mathrm{with}\ \mathrm{matching}\ \mathrm{entries}\ \mathrm{in}\ \mathrm{both}\ \mathrm{primary}\ \mathrm{data}\ \mathrm{tool}\ \mathrm{and}\ \mathrm{MCTS}\ \mathrm{porta}{\mathrm{l}}_{\mathrm{j}}}{\mathrm{Total}\ \mathrm{no}.\ \mathrm{of}\ \mathrm{common}\ \mathrm{data}\ \mathrm{fields}\ \mathrm{filled}\ \mathrm{in}\ \mathrm{both}\ \mathrm{primary}\ \mathrm{data}\ \mathrm{tool}\ \mathrm{and}\ \mathrm{MCTS}\ \mathrm{porta}{\mathrm{l}}_{\mathrm{j}}}\times 100$
Overall System Performance		
6	Percentage of all possible MCTS portal data fields for sampled beneficiaries with accurate entries	$\frac{\mathrm{Total}\ \mathrm{no}.\ \mathrm{MCTS}\ \mathrm{portal}\ \mathrm{data}\ \mathrm{f}\mathrm{ields}\ \mathrm{with}\ \mathrm{entries}\ \mathrm{that}\ \mathrm{match}\ \mathrm{the}\mathrm{ir}\ \mathrm{counterpart}\ \mathrm{entries}\ \mathrm{in}\ \mathrm{the}\ \mathrm{primary}\ \mathrm{data}\ \mathrm{sourc}{\mathrm{e}}_{\mathrm{j}}}{\mathrm{Total}\ \mathrm{MCTS}\ \mathrm{portal}\ \mathrm{data}\ \mathrm{f}\mathrm{ields}\ \mathrm{f}\mathrm{o}\mathrm{r}\ \mathrm{whole}\ \mathrm{sampl}{\mathrm{e}}_{\mathrm{J}}}\times 100$

_i_ : FHW Register or MCTS portal

_j_ : Pregnant Women or Children

The data found in FHW field registers were considered the primary data source for assessing MCTS portal data accuracy, as these are the rawest form of beneficiary and service delivery data. FHWs in Rajasthan used the MCH register, while those in UP used the MCTS register as their primary data recording tools. The DQA considered 20 data fields for pregnant women, and 19 data fields for children (see Tables 3 and 4). For DQA indicators which entailed comparing only data fields found in both the FHW field register and the MCTS portal, these ‘common fields’ were used for assessment (refer to Tables 3 and 4).

**Table 3:** Data fields for DQA, pregnant woman

Name of data fields used for DQA	Data fields found in each source				Common data fields in both sources	
	Y = Yes/N = No					
	Uttar Pradesh		Rajasthan		Uttar Pradesh	Rajasthan
	Primary data source	State MCTS portal	Primary data source	State MCTS portal	Primary source and state MCTS portal	
Name	Y	Y	Y	Y	Y	Y
Address	Y	Y	Y	Y	Y	Y
Husband Name	Y	Y	Y	Y	Y	Y
Mob No.	Y	Y	Y	Y	Y	Y
Date of Birth/Age	Y	Y	Y	Y	Y	Y
JSY Beneficiary	Y	Y	N	Y	Y	N
LMP	Y	Y	Y	Y	Y	Y
1st ANC Date	Y	Y	Y	Y	Y	Y
2nd ANC Date	Y	Y	Y	Y	Y	Y
3rd ANC Date	Y	Y	Y	Y	Y	Y
4th ANC Date	N	Y	Y	Y	N	Y
TT 1 Date	Y	Y	Y	Y	Y	Y
TT 2 Date	Y	Y	Y	Y	Y	Y
Date of delivery	Y	Y	N	Y	Y	N
Place of delivery	Y	Y	N	Y	Y	N
Date of JSY benefit payment	Y	Y	Y	Y	Y	Y
Outcome of current pregnancy	Y	Y	N	Y	Y	N
Weight of child	Y	Y	N	Y	Y	N
Child sex	Y	Y	N	Y	Y	N
PNC Home Visit	Y	Y	N	Y	Y	N
Total	19	20	13	20	19	13

**Table 4:** Data fields for DQA, children

Name of field on DQA done	Data fields found in each source				Common data fields in both sources	
	Y = Yes, N = No					
	Uttar Pradesh		Rajasthan		Uttar Pradesh	Rajasthan
	Primary data source	State MCTS portal	Primary data source	State MCTS portal	Primary source and state MCTS portal	
Name	Y	Y	Y	Y	Y	Y
Mother/ Father Name	Y	Y	Y	Y	Y	Y
Phone No.	Y	Y	N	Y	Y	N
Date Of birth	Y	Y	Y	Y	Y	Y
Place of delivery	Y	Y	N	N	Y	N
Caste	Y	Y	Y	N	Y	N
Gender	N	Y	Y	Y	N	Y
BCG	Y	Y	Y	Y	Y	Y
OPV0	Y	Y	N	Y	Y	N
HepB0	N	Y	N	Y	N	N
DPT1	Y	Y	Y	Y	Y	Y
OPV1	Y	Y	Y	Y	Y	Y
HepB1	Y	Y	Y	Y	Y	Y
DPT2	Y	Y	Y	Y	Y	Y
OPV2	Y	Y	Y	Y	Y	Y
HepB2	Y	Y	Y	Y	Y	Y
DPT3	Y	Y	Y	Y	Y	Y
OPV3	Y	Y	Y	Y	Y	Y
HepB3	Y	Y	Y	Y	Y	Y
Total	17	19	15	17	17	14

An assessment survey, comprising semi-structured questionnaires, survey investigator field notes and observation checklists, was used to document the implementation dynamics of the MCTS. The following were the factors covered under the assessment survey:

- Human resources (HR) and infrastructure
- Budget and expenditure
- Beneficiary estimation and identification
- Service delivery tools and utilization
- Monitoring and supervision

Service delivery staff at the peripheral levels and supervisory and administrative staff up until the district levels were interviewed. Table 5 presents the staff interviewed in each state at each level of the health system, and the tools used for data recording.

**Table 5:** List of assessment survey participants

			Actual sample		
Level	Health infrastructure	Key informants/Area	Rajasthan	Uttar Pradesh	Tools
District	District Health Society. Chief Medical Officer’s office	District Immunization Officer (DIO)	1	2	Semi Structured questionnaire
		Management of Information System (MIS) Officer/Monitoring & Evaluation (M&E) Officer.	2	2	
		HR capacity building and infrastructure	2	2	Observation checklist
Block	Block Primary Health Centre (Data Entry Point for MCTS)	Medical Officer in charge (MOIC)	4	4	Semi-Structured questionnaire
		Block Program Manager (BPM)	2	4	
		Data Entry Operator (DEO)	4	4	
		HR capacity building and infrastructure	4	4	Observation checklist
Sub-Block	Health Sub-Centre	Auxiliary Nurse Midwife (ANM)	7	8	Semi structured questionnaire
		Accredited Social Health Activist (ASHA)	7	8	
		Immunization session/Village Health Nutrition Day (VHND)	8	8	Observation checklist

Position of DIO and two BPMs in one Rajasthan district were vacant. One ANM and one ASHA from Rajasthan could not be interviewed as the Village Health and Nutrition Day was scheduled in only one health facility of the assessed block on a particular day

### Ethics framework and data handling

The Institutional Ethics Committee (IEC) within the Public Health Foundation of India (PHFI) granted ethical approval for the assessment methodology. Before administering the semi-structured questionnaires and filling in the information in the DQA tools, all research participants were presented with the request letter from the Ministry of Health that explained the purpose of the assessment.

An informed consent form was not presented, as the study participants, who were all government health workers, participated in the study upon the request of the Ministry of Health. That said, survey investigators assured participants of the confidentiality of their responses, and that all presentations of data and analyses would be anonymized to protect their identities, before administering the study instruments.

Adhering to the ethical framework, the research team anonymized and delinked the primary data from individual names, or other easily discernible identities. This ensured that the data and the presentation of analyses could not be tracked to unique sources.

For ensuring data storage safety, access to the primary data, which included hard copies of the semi-structured questionnaires, field notes and the DQA tools, was restricted to three personnel within the study team as approved by the PI of the study. These three personnel also have exclusive access to the digitized version of the data.

As the MCTS is an information system for government health operations, it's data is password protected and is not openly available. The study team accessed MCTS data with the approval of the Ministry of Health.

Quantitative and qualitative data captured by the study team’s semi-structured questionnaire can be shared by the study team upon request.

## Results and discussion

### MCTS performance

The DQA overall performance results were 34 % for sampled pregnant women, and 33 % for sampled children in the Rajasthan study areas. In the UP study areas, the results were 18 % for sampled pregnant women, and 25 % for sampled children (Table 2, indicator 6).

### MCTS data completeness

Low data completeness rates were the primary factor leading to these poor performance numbers. While the Rajasthan sample had the higher MCTS beneficiary registration rates out of the two assessed states (all sampled women, and 85 % of sampled children had their profiles registered in the MCTS portal), the completeness of these profiles was 64 % for both sampled pregnant women and children (Table 2, indicators 1 and 2).

In the UP sample, 21 % of sampled pregnant women and 43 % of sampled children were found not to have MCTS profiles (Table 2, indicator 1). The registered profiles in the MCTS portal were 38 % complete for sampled pregnant women and 56 % complete for sampled children (Table 2, indicator 2).

### Primary data source completeness

One reason for incomplete MCTS portal data is the incompleteness of the primary data source. In Rajasthan, the primary data source (MCH Register) from which data is entered into the MCTS portal was 78 % complete for sampled pregnant women and 88 % complete for sampled children (Table 2, indicator 2). In UP, primary data source (MCTS Registers) completeness was 58 % for sampled pregnant women and 73 % for sampled children (Table 2, indicator 2).

In both states, the primary data sources were not designed or standardized to fully match the data needs of the MCTS portal. In Rajasthan, the MCH register (primary data source), when fully filled, met 65 % of the MCTS portal’s data needs for pregnant women, and 82 % for children. In UP, there was a lack of linguistic standardization in the names of data fields between the MCTS register (primary data source) and the portal. For example, the birth dose of the Hepatitis B vaccine was recorded as “Hep B1” in the register, while it was labelled “Hep B0” in the portal. These discrepancies can compromise data quality.

### MCTS data accuracy

Accuracy rates in Rajasthan and UP for data on sampled pregnant women were at 86 %, and 79 % respectively. Accuracy rates for data on sampled children were at 92 % for UP and 71 % for Rajasthan (Table 2, indicator 5).

Though data accuracy rates may be high in both assessed states, the denominator quantity of data on which this was measured was too small for it to have an impact on overall data quality performance. Highly incomplete data, regardless of accuracy rates, compromise effective service delivery planning and beneficiary tracking.

**Fig. 2:**
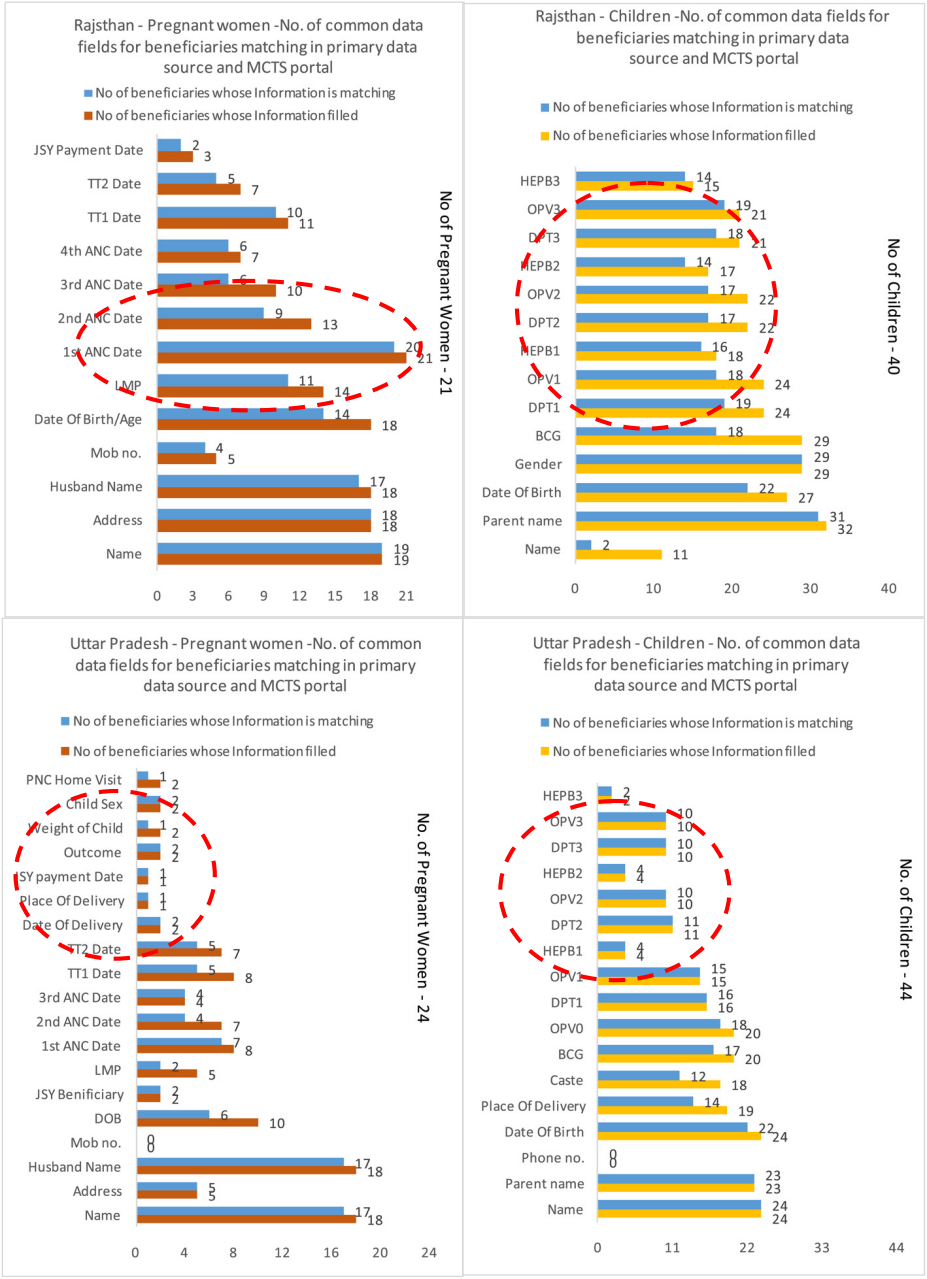
No. of common data fields matching in primary data tool and MCTS portal, Rajasthan, Uttar Pradesh

In Rajasthan, data accuracy for Tetanus Toxoid (TT) 1 and TT2 was 91 and 71 % respectively. However, TT1 vaccination data was available for 11 pregnant women, while TT2 vaccination was available for seven pregnant women only, out of 21 sampled. In Rajasthan, data accuracy for Hep B1, 2 and 3 was 89, 82 and 93 % respectively. However, the highest data availability for Hep B vaccination details was for the Hep B1 dose, which was only 18 out of 40 sampled children. In UP, data accuracy for Hep B1, 2 and 3 was 100 %; however, out of the 44 children sampled, the details of only two to four were filled in the portal (Fig. 2).

In Uttar Pradesh, qualitative responses from data entry personnel indicated that data fields such as beneficiary phone number were mandatory in the MCTS portal, and thus in the absence of this data, DEOs were compelled to enter the contact details of the relevant FHWs instead. These same qualitative responses also indicated that in cases of unavailable Oral Polio Vaccine dose information at birth (OPV0), DEOs carried forward Bacillus Calmette Guerin (BCG) vaccination dates for 0PV0, based on the assumption that BCG and OPV0 are provided to a child on the same day, which may not be the case for a variety of reasons (vaccine shortages, poor training of health workers). Such assumptions can compromise data accuracy.

### Challenges of data transfer from primary data source to the MCTS portal

**Fig. 3:**
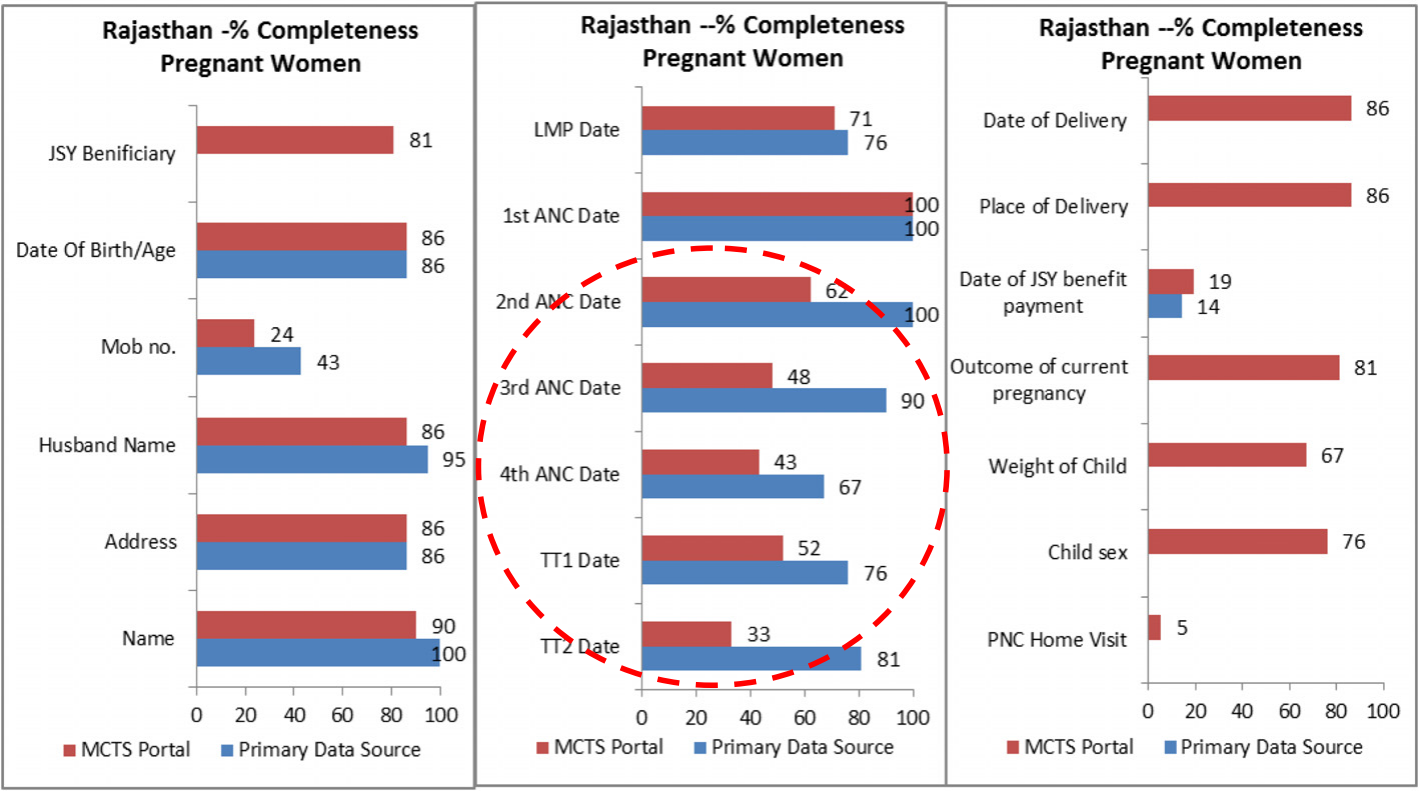
% completeness, pregnant women, Rajasthan

**Fig. 4:**
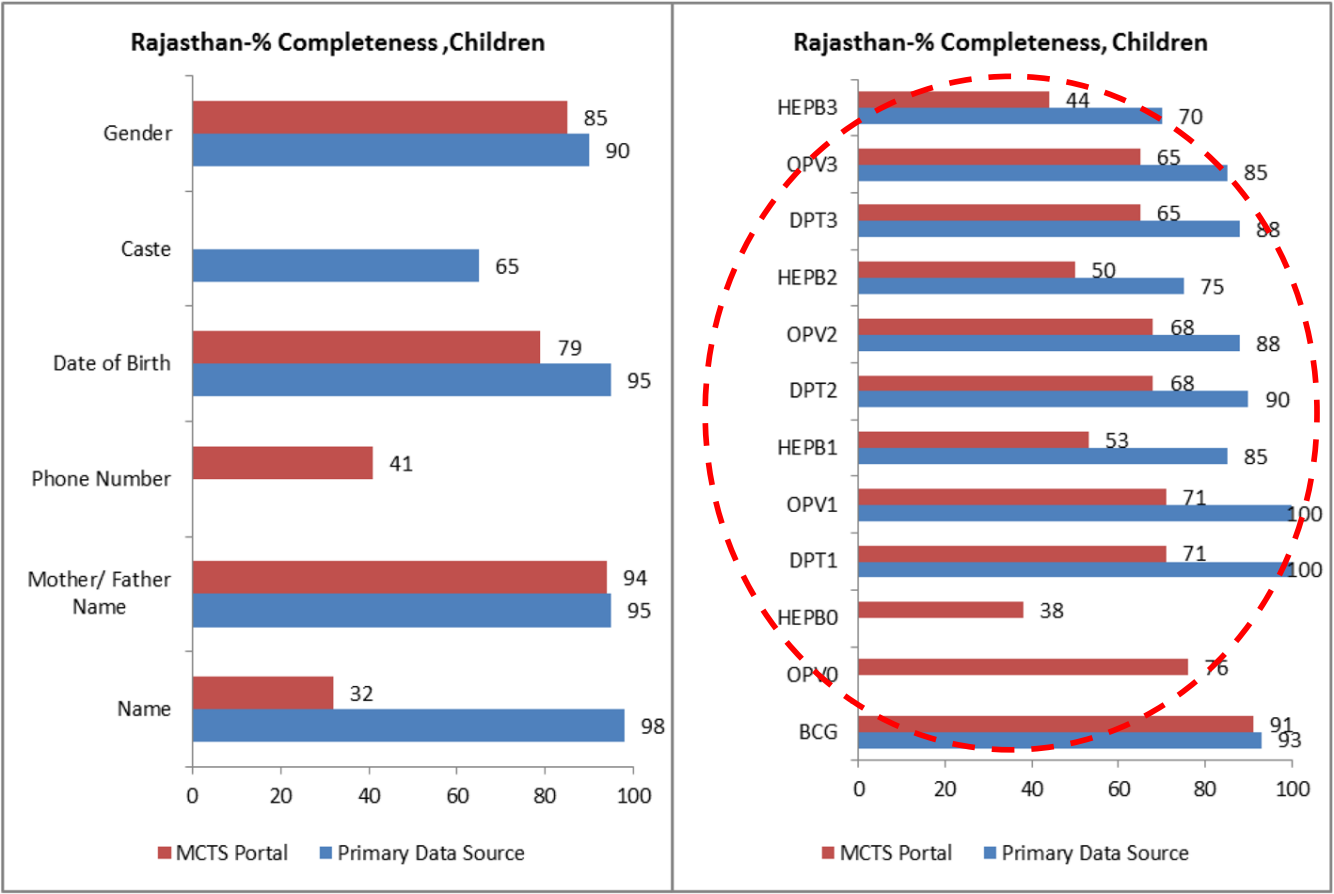
% completeness, children, Rajasthan

**Fig. 5:**
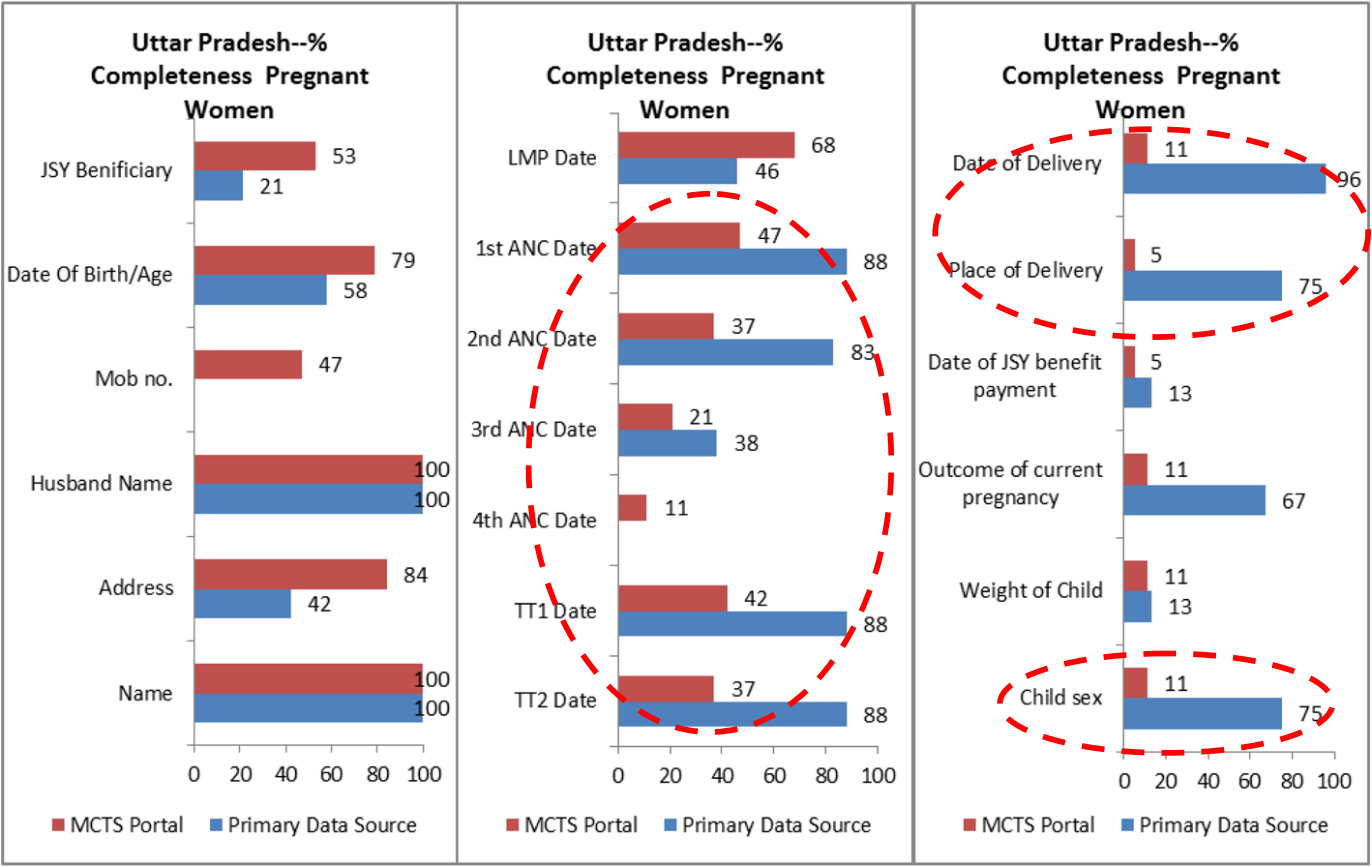
% completeness, pregnant women, Uttar Pradesh

**Fig. 6:**
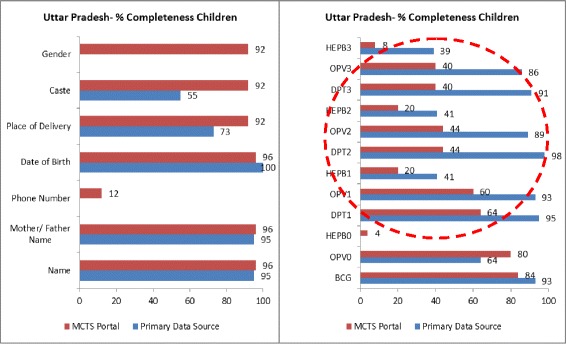
% completeness, children, Uttar Pradesh

Data field-wise completeness rates in the study areas of Rajasthan and UP (Figs. 3, 4, 5, and 6) indicated that service delivery data collected by field staff was often incompletely transferred to the MCTS portal. In both states, a comparison of data fields such as ANC and TT vaccination details for pregnant women and DPT (1, 2 and 3), OPV (1, 2 and 3), HEPB (1, 2 and 3) for children (all services delivered routinely at the field level) demonstrated much higher completeness rates in the primary data source than in the MCTS portal.

The root causes of these data quality weaknesses lay in suboptimal field level data collection, consolidation and transfer processes, inconsistent training levels for health staff and a lack of clear monitoring and supervision guidelines.

### Data collection, consolidation and transfer

#### New beneficiary identification

The process for identifying new MCH beneficiaries, carried out by FHWs, should ideally be robust enough to capture new beneficiary details and transfer them to the portal sufficiently rapidly so that the MCTS can produce better forecasts of future service delivery needs. Frequent meetings between FHWs to consolidate new beneficiary details should form the basis of new beneficiary identification practices. These meetings should include Auxiliary Nurse Midwives (ANMs), who are the FHWs delivering services, and Accredited Social Health Activists (ASHAs), the FHWs who reside in villages and mobilize the local community to access services.

In Rajasthan, five out of seven interviewed ASHAs reported meeting ANMs for data sharing in gaps of more than one week (four reported once a month, and one once a fortnight). In UP, out of eight ASHA responses on frequency of data sharing meetings with ANMs, two reported once a week, three once in a fortnight, two once a month, and one reported meeting ANMs on immunization session days (twice weekly). On the other hand, three out of four interviewed data entry personnel stated that they receive new beneficiary details once a month, while all interviewed ANMs reported assisting with data entry once a month. In UP, vaccination sessions normally occur twice a week in each ANM’s catchment area, so this long gap in the registration of new beneficiaries into the MCTS compromises its function as a tracking and service delivery planning tool.

#### Data recording tools

In all surveyed areas, there was an absence of standardization in the data tools, and data processes. Assessment survey data revealed that ANMs transferred field information from their main registers onto hand-drawn formats to be transferred for data entry. Data from UP highlighted a shortage of MCTS registers at the sub-district level (two out of four surveyed blocks). In the UP study areas, apart from MCTS registers (primary data source), ANMs used their diaries, local formats and tally sheets for recording service delivery information during immunization sessions. In the Rajasthan study areas, apart from the MCH register (primary data source), ANMs maintained hand-drawn registers to record MCTS-related information. Six out of eight ANMs in UP, and at least three out of seven interviewed ANMs in Rajasthan reported using a format for data transfer that was different from that used for field-level data recording. The use of multiple data recording and transfer tools resulted in duplication of data documentation work for FHWs.

This additional burden of documentation imposed by the MCTS on the ANM, who is already charged with a range of other record-keeping duties, detracts from the MCTS’ ideal role as a service delivery facilitator. Moreover, the additional inconvenient layer of manual data recording may compromise the completeness and accuracy of data transferred into the portal.

#### MCTS workplans

The MCTS has developed an inbuilt mechanism for generating a due list of beneficiaries before each immunization session. The e-due list is called the MCTS workplan. Assessment data highlighted minimal use of the MCTS workplan. Only two out of seven interviewed ANMs in Rajasthan reported that they shared the workplans with ASHA workers who are responsible for mobilizing beneficiaries. In UP, six out of eight interviewed ANMs had never received workplans. Additionally, qualitative evidence highlighted challenges in the completeness and accuracy of MCTS workplans.

### Training

Assessment survey evidence also indicated that MCTS training among service delivery, supervisory and data entry staff was inconsistent.

In Rajasthan, all three interviewed ANMs in one district reported not having received MCTS training, while all four in the other district reported having received it. ANMs themselves shared areas in which they needed greater training, most of which are crucial to the optimal functioning of the MCTS: using recording tools and computer-generated workplans, and refresher trainings. Qualitative feedback from data entry staff (District Immunization Officers and Management Information Systems officials) and supervisory officials (DIOs) also emphasized the need for MCTS training for FHWs.

In UP there was a shortage of MCTS-trained staff, with the exception of data entry personnel, in the assessment areas. However, two DEOs reported that they did not find the current training regimen sufficient. Importantly, none of the interviewed block level supervisory officials had received MCTS training, while all but one interviewed ANM had also not been trained. Considering that block level officials and FHWs are the crucial link between beneficiaries and the MCTS, the lack of trained personnel in this area is a major weakness.

### Monitoring and supervision

#### Supervisory engagement with the MCTS at the block level

Assessment survey data highlighted that while MCTS performance was discussed to varying degrees by block supervisors with FHWs during routine monitoring meetings, the engagement of these supervisors with the MCTS and its outputs needed improvement. All block level supervisory officials in Rajasthan, and at least one supervisory official in each surveyed block in UP, reported discussing MCTS implementation issues with FHWs (ANMs and ASHAs) during monitoring and supervision meetings. Three out of four surveyed blocks in Rajasthan had at least one supervisory official (Block Program Manager/Medical Officer In-charge) directly using the MCTS web portal. In UP none of the interviewed supervisory officials used the MCTS web portal. All surveyed blocks in Rajasthan and two out of the four surveyed blocks in UP used reports and data generated by the MCTS to guide MCH program management and to prepare monthly progress reports. The lack of training for supervisory officials could be a key reason behind the lack of consistent engagement between supervisory staff and the MCTS.

#### Supervision and feedback processes from the district and state levels

The feedback that block level officials in both surveyed states received from higher levels on MCTS implementation was not delivered in any standardized form. In Rajasthan, out of the four interviewed MOICs, one reported getting feedback over email, two at review meetings, and one did not specify. Out of the two interviewed BPMs, one mentioned review meetings, and both identified e-mail. MOICs and BPMs in UP reported a combination of district meetings, e-mails, and supervisory visits being used by higher officials for sending feedback. Two MOICs explicitly stated that there was no structured feedback mechanism among supervisory officials for MCTS implementation.

### Use of ICT in the MCTS

While the MCTS has a mobile component through which the system responds to health staff and beneficiaries (which was not a primary assessment component of this study), our limited survey evidence suggests that the engagement of health staff with this component could be improved. Out of all surveyed supervisory officials, only one MOIC in Rajasthan, and two officials in UP (one MOIC and one BPM) had their mobiles registered in the MCTS and received SMSs or other communication. The case was similar for four out of seven ANMs in Rajasthan, and six out of eight ANMs in UP.

### HR and infrastructure

Irregular electricity supply, inconsistent internet connectivity and the slow speed of the MCTS web portal were some of the challenges faced by block-level facilities, which act as the primary MCTS data entry points. Two out of four surveyed blocks in Rajasthan, and three out of four in UP highlighted irregular power supply. Three out of four surveyed blocks in Rajasthan and two out of four surveyed blocks in UP faced inconsistent internet connectivity.

In UP, interviewed staff pointed out HR problems that compromised MCTS implementation. Among the issues highlighted were the lack of dedicated data entry personnel, irregular receipt of their salaries and breaks in their contract renewal process. In Rajasthan, interviews highlighted issues of work burden for data entry personnel, as MCTS data entry was an additional charge imposed on existing staff with other responsibilities.

## Conclusions

The MCTS implementation challenges outlined here may mirror some of the hurdles that similar initiatives face in other LMICs. In order for HISs such as the MCTS to play a truly catalysing role in the delivery of MCH services, the following fundamentals need to be adequately addressed in order to improve MCTS performance.

Standardized data tools and processes must be clearly defined. Firstly, linguistic standardization in the names given to data fields should be observed, not just within the MCTS, but also with other important HISs, such as India’s Health Management Information System. This would improve interoperability between the MCTS, which is primarily meant to aid FHWs with beneficiary-specific service delivery needs, and the HMIS, which is a monitoring MIS, collecting aggregate monthly service delivery data from health centres. This would be a first step in reducing the observed inconsistencies between the data in the MCTS and the HMIS. Improved standardization would also reduce confusion among FHWs in recording and transferring data as two data fields meant to be operationally similar would be called by the same name.

Secondly, registers and formats should be standardized to meet the needs of the MCTS portal and the service delivery needs of FHWs. The design of these registers and formats should be as intuitive as possible, and take special care to minimize data work for FHWs.

Thirdly, to complement the aforementioned standardized registers and formats, clearly defined standardized data processes and guidelines need to be designed for staff at the most peripheral levels of the health system. These guidelines should clearly lay out a plan for data collection, consolidation, and transfer to the data entry point, with stipulated timelines. It is also crucial that the creation of these data processes are informed, as much as possible, by existing service delivery timelines and norms so that they can complement the obligations of healthcare staff. Taking full cognisance of existing healthcare workflows in the planning and implementation of a system like the MCTS is pivotal in realizing organizational efficiency gains [8, 18–20], in addition to garnering sufficient buy-in amongst FHWs for e-health innovations [21].

ICT innovations that are better conceptualised and implemented could potentially be used to ease the workload of FHWs. For example, the State of Karnataka in South India has experimented with additional innovations in the use of mobile phones for FHWs [22], and there have been other activities and discussions on the integration of ICT into HISs both in India [21, 23, 24] and in other LMICs [25, 26]. Existing ICT innovations for HISs should be thoroughly investigated to glean implementation lessons and provide a basis for evaluating potential technologies.

Guidelines for monitoring and evaluating the implementation of the MCTS should also be defined for supervisory officials, primarily at the block and district levels. These guidelines should include dashboard indicators to measure implementation progress, define structured feedback mechanisms between supervisory staff at different levels of the health system, provide guidance on conducting rapid DQAs to assess data quality [27] and define methods for utilizing collected data for health program management and evaluation. Consistent data quality oversight and data usage by supervisory officials are crucial for HISs to function well [10, 28–30], so supervisors need to respond actively to extant data for health workers to prioritize data quality. The supervision approach should also be supportive and not punitive in nature, so that health workers in peripheral facilities are encouraged to be frank about their weaknesses and are open to learning [31].

Once these protocols and guidelines are defined, all relevant staff in the health system should be adequately trained in their use. In India, the National Institute of Health and Family Welfare (NIHFW) has developed an overarching HIS training guide, which contains a brief module on the MCTS. This module should be tailored to the needs and interactions of each category of health staff with the MCTS. Supervisory officials, who need to generate reports and oversee implementation, would interact with the MCTS very differently from FHWs, who are responsible for field data processes and respond to service delivery planning outputs from the system.

The pedagogy of the training programs must also emphasize the value of data quality in improving not only the performance of health programs, but also the working lives of health workers. Refresher trainings, informed by ongoing implementation weaknesses, should be built into the training plan. The potential of e-learning strategies in enhancing continuous learning among health workers should be explored. E-learning can ensure direct and consistent capacity building communication with health workers [32]. These integrative and capacity building activities are essential not just for the MCTS, but for any HIS that seeks to facilitate and support the work of public health staff [8].

If the establishment of the MCTS has led to increased workload for administrative personnel, such as data entry staff, provisions should be made to hire needed staff in order to ensure that the system functions smoothly. An adequate supply of consumables, especially data collection and consolidation registers for FHWs, need to be ensured, while reliable internet connectivity is important for timely data entry into the MCTS portal. For resource-poor areas in which power supply and internet connectivity are chronic problems, offline data entry capabilities need to be built into the MCTS web portal, so that the work flow of data entry personnel is not abruptly interrupted.

The implementation process of the MCTS should also be reoriented to be as least disruptive as possible to existing processes and workflow patterns. MCTS workplans were envisioned to replace existing service delivery planning tools among FHWs and block officials, and while replacing the plethora of planning tools in India with a standard format was a laudable idea, doing so without first improving data quality would lead to severe disruptions in MCH service delivery. As such, the first priority should be to improve MCTS data reporting and quality, before insisting that MCTS workplans replace existing planning tools. This evolutionary, rather than revolutionary, approach is important to the success of such interventions in India [33], and potentially in other LMICs.

Though these findings may not be representative of the MCTS’ performance in India as a whole, or in Rajasthan or UP as a whole, they do highlight the kinds of challenges that HISs face in resource-poor settings. Under India’s National Rural Health Mission (NRHM), districts are the primary planning nodes for the delivery of health care services, so the selection of two districts each in two states with traditionally poor health indicators, and the collection of DQA and survey data down to the most peripheral health units within these districts, was meant to provide a picture of HIS implementation challenges that may also be prevalent in locations with similar resource constraints.

Our findings indicate that larger-scale studies covering more districts, and potentially whole states, would be useful in measuring the performance of HISs in India. In particular, our study does not address the impact that the MCTS has had on public health service delivery. This is an important area of focus, as the ultimate goal of HISs is to inform and support health systems to better meet the needs of beneficiaries. This study also does not substantially grapple with questions on how and when to deploy IT innovations to improve HIS performance. Greater research into appropriate deployment strategies for new technologies and impact assessments of existing technologies for HISs would be of high importance in informing HIS policy in resource-poor settings.

## Endnote

^1^The MCTS is called the PCTS (Pregnancy, Child Tracking, and Health Services Management System) in Rajasthan, but for the sake of consistency we have used “MCTS” throughout this manuscript. The PCTS was launched in 2008, slightly earlier than the national MCTS. The two systems have converged.

## Abbreviations

ANC: Ante-Natal Checkup
ANM: Auxiliary Nurse Midwife
ASHA: Accredited Social Health Activist
BCG: Bacillus of Calmette Guerin
BPM: Block Program Manager
DEO: Data Entry Operator
DIO: District Immunization Officer
DPT: Diphtheria Pertussis Tetanus
DQA: Data quality assessment
FHW: Frontline health worker
GoI: Government of India
HepB: Hepatitis B
HIS: Health information system
HMIS: Health management information system
HR: Human resource
HSC: Health Sub-Centre
ICT: Information communication technology
IDQA: Immunization data quality assessment
IEC: Independent Ethics Committee
LMIC: Low and Middle Income Countries
MCH: Maternal and child health
MCTS: Mother and child tracking system
MDG: Millennium development goals
MIS: Management of information systems
MMP: Mission Mode Project
M&E Officer: Monitoring and Evaluation Officer
MoHFW: Ministry of health and family welfare
MOIC: Medical Officer In-Charge
NeGP: National e-Governance Plan
NRHM: National Rural Health Mission
OPV: Oral Polio Vaccine
PCTS: Pregnant women and child tracking system
PHC: Primary Health Center
PHFI: Public Health Foundation of India
PNC: Postnatal care
SMS: Short message service
UIP: Universal Immunization Program
UP: Uttar Pradesh
VHND: Village health and nutrition day
WHO: World Health Organization
